# Kardiogenetik in Deutschland – ein (Rück‑)Blick

**DOI:** 10.1007/s00399-024-01008-y

**Published:** 2024-02-28

**Authors:** E. Schulze-Bahr

**Affiliations:** Institut für Genetik von Herzerkrankungen (IfGH), Spezialambulanz für Patienten mit genetischen Herzerkrankungen, Universitätsklinikum Münster (UKM), Domagkstr. 3, 48145 Münster, Deutschland

**Keywords:** Kardiogenetik, Ionenkanalerkrankungen, Geschichte, Sinusknotendysfunktion, Erregungsleitungsstörung, Cardiogenetics, Ion channel diseases, History, Sinus node dysfunction, Cardiac conduction disease

## Abstract

Die Entwicklung des Kardiogenetik in Deutschland hat seit der Mitte der 90er Jahre eine zunehmende Entwicklung mit vielen eigenen, zum Teil wichtigen und wegweisenden Beiträge. Ausgangspunkt war und ist immer noch der Patient mit seiner Familie, z. B. mit einer familiären Arrhythmieform oder einer Kardiomyopathie, die Aufklärung der genetischen Ursache und die personalisierte Behandlung der Betroffenen.  Das wissenschaftliche, immer transnational orientierte Interesse, ein ursächliches Gen zu identifizieren und den zugrundeliegenden Pathomechanismus aufzudecken, hat beim Brugada-Syndrom, Kurzen QT-Syndrom und Erregungsleitungsstörung oder Sinusknotendysfunktion, aber auch bei DCM oder ARVC zu beachteten Beiträgen geführt. Wichtig ist jedoch der Weg zurück (bench > bed side): Implementierung von nationalen wie internationalen Empfehlungen zur kardiogenetischen Diagnostik in die kardiologische Versorgung und die personalisierte Betreuung und Therapie Betroffener.

## Abstract (en)

The development of the cardiogenetics field in Germany has been increasing since the mid-1990s with many national contributions, some of them were really important and groundbreaking. The starting point was and still is the patient and his family, e.g. with a familial form of arrhythmia or cardiomyopathy, the clarification of the genetic cause and the personalized treatment of those being affected. The scientific, always translationally oriented interest in identifying a causative gene and uncovering the underlying pathomechanisms has led to notable contributions for Brugada syndrome, short QT syndrome and cardiac conduction disorders or sinus node dysfunction, but also in DCM or ARVC. What is important, however, is always the way back (bench > bed side): implementation of national and international recommendations for cardiogenetic diagnostics in daily cardiological routine and the personalized care and therapy of those being affected.

## *Rückblick*: Familiäre (Erst‑)Beschreibungen von Arrhythmien und Kardiomyopathien – ein Schlüssel von der idiopathischen Erkrankung zur Genidentifizierung

Das familiär gehäufte Auftreten einer seltenen, aber durchaus auch einer häufigeren Herzerkrankung (z. B. KHK) ist und bleibt für die betroffenen Familien etwas Besonderes, aber auch für die behandelnden Ärzte mitunter etwas Spezielles. Einerseits kommt bei Betroffenen (und auch Ärzten) die Frage nach dem „Warum?“ auf, andererseits haben die Nichtbetroffen oft die Frage nach dem „Ich auch?!“ Mittlerweile verfestigt sich, auch in allen aktuellen Leitlinien, immer mehr die anamnestisch wichtige Frage beim Indexpatienten „Wer noch in der Familie?“, mit allen primärpräventiven Intentionen. Die Kardiogenetik kann hier zunehmend durch die Möglichkeit der Früherkennung bzw. Nachweis der sog. Merkmalsträgerschaft (Genträger ohne aktueller Krankheitspenetranz) wichtige klinische Informationen für die Beratung und Behandlung der Familien bereitstellen.

**Figure 1:**
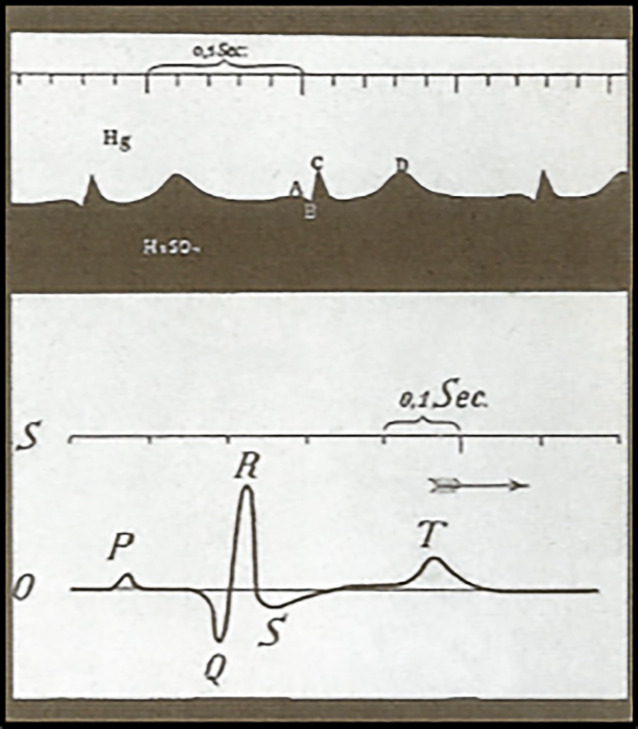

Das familiäre Auftreten einer Erkrankung war früher bei der Entdeckung, dass „etwas familiär gehäuft auftritt“, etwas Fabel- oder Anekdotenhaftes, was jedoch auch in deutschen Sprachraum bzw. von einzelnen Kardiologen als solches publiziert wurde. Für familiär auftretende Arrhythmieformen wurde dieses im Weiteren natürlich durch die Entwicklung der verbesserten Registrierung der elektrischen Aktivität des Herzens (Abb. 1) begünstigt – obgleich EKG-Registrierungen heutzutage eigentlich „Standard wie Alltag“ sind, wird durch die familiären Arrhythmiesyndrome unser kardiologischer Blick auf das EKG immer wieder erneut geschärft – sei es beim frühen Repolarisationssyndrom („early repolarisation syndrome“, ERS) oder beim sog. Bundgaard-Syndrom („familial ST-segment depression“; [13]).

**Figure 2:**
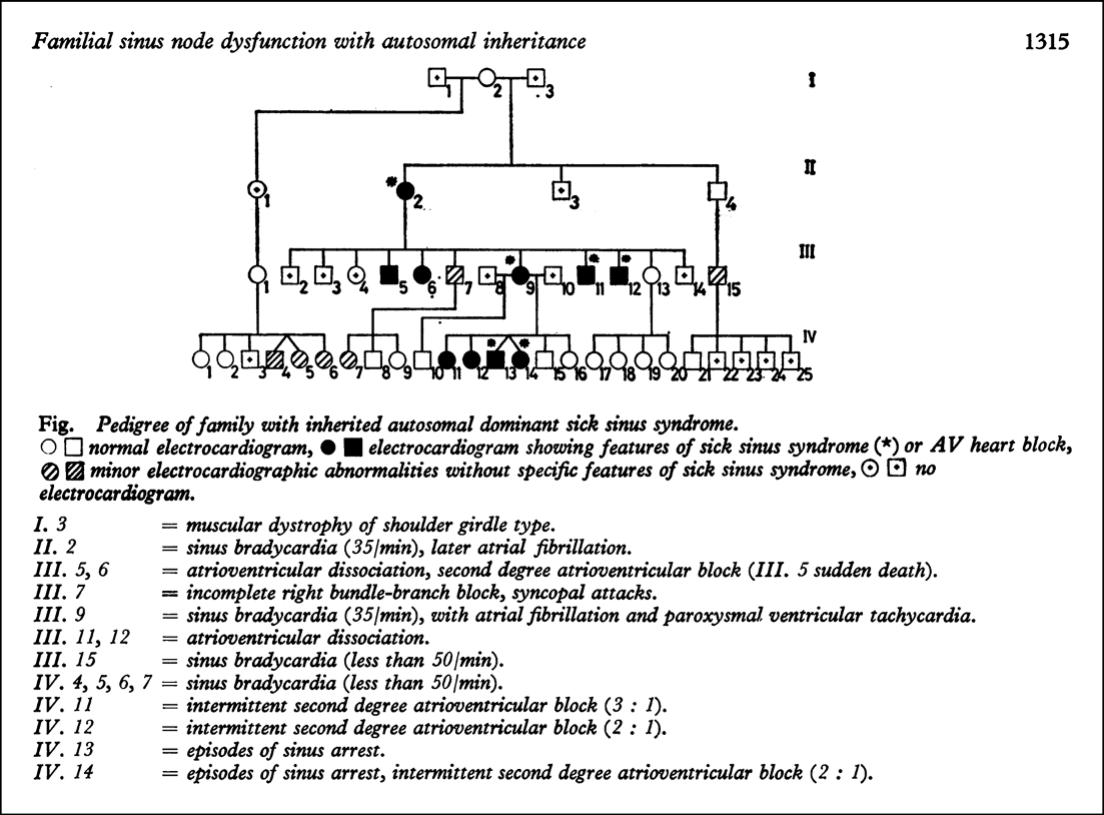

Initial wurden Beobachtungen über familiär vorkommende Herzrhythmusstörungen als sehr selten angesehen, vor allem weil in älteren altere Veröffentlichungen die erforderlichen elektrokardiographischen Befunde nicht als hinreichend eingestuft wurden [73]. So beschrieben Böhm und Lang 1962 eine große Mehrgenerationfamilie [9] mit „… *multifokaler Kammerextrasystolie … Ein sicherer Kausalzusammenhang zu einer infektiös-toxischen oder degenerativen Myokarderkrankung, einem angeborenen oder erworbenen Herzfehler, dem ebenfalls familiär gehäuft auftretenden Krankheitsbild der idiopathischen Kardiomegalie, einer Störung des Fettstoffwechsels und anderen Organerkrankungen lag nicht vor. Pathogenese und Ätiologie der beschriebenen familiären Herzrhythmusstörung bleiben … ungeklärt*“. Spang beschrieb 1957 eine konstitutionelle Form der Sinusbradykardie bei offensichtlich gesundem Herzen und chronotroper Inkompetenz und zitierte eine Beobachtung von Wenckebach, der ebenfalls ein familiäres Vorkommen in seiner eigenen Familie beobachtet hatte („… *In der Familie Wenckebach sind z.* *B. Vater, Mutter und 3 von 4 Kindern Bradykardiker. Ihre Pulsfrequenz fiel in der Jugend bisweilen unter 40; alle sind zu gesunden, kräftigen Menschen herangewachsen …*“; [73]). Ein weiteres Beispiel für eine autosomal-dominant vererbte Form der Sinusknotendysfunktion und zum Teil AV-Blockierung wurde 1978 von den Kieler Kollegen Lehmann und Klein veröffentlicht (Abb. 2).

Die Familie fiel Jahrzehnte nach der Originalveröffentlichung im Rahmen der Bradykardiebehandlung von Nachkommen erneut auf und einige Familienmitglieder konnten für Forschungsuntersuchungen aktiviert werden; nach Durchführung einer Gesamt-Exom-Sequenzierung wurde zwischenzeitlich eine kausale Genvariante identifiziert und molekularbiologisch charakterisiert (Schulze-Bahr und Team, unveröffentlicht; 2024). Die Dissektion pathophysiologischer Komponenten und Mechanismen zur initial idiopathischen Sinusknotendysfunktion soll dabei weitere Türen für eine Nicht-Device-Therapie zur Sinusknotenaktivierung perspektivisch ermöglichen.

Neben den wenigen, aber charakteristischen Familien mit einem nachvollziehbaren Erbgang und einem isolierten, kardialen Phänotyp wurde zudem zunehmend aus syndromalen, insbesondere neurologischen Erkrankungen klar, dass es eine kardiale Mitbeteiligung (überwiegend atrioventrikuläre Erregungsleitungsstörungen und Kardiomyopathien) geben kann; so veröffentlichten z. B. in einer eigenen Fallserie und einer Zusammenstellung der bisherigen, veröffentlichten Fälle von Friedreich-Ataxie kardiale und EKG-Befunde („*Auffälligkeiten der P‑Zacke, Zeichen ventrikulärer Seitenbelastung, Störungen der Repolarisationsphase des EKGs“*), was zum Teil der Erstbeschreiber Friedreich auch schon vereinzelt bemerkte [29].

**Figure 3:**
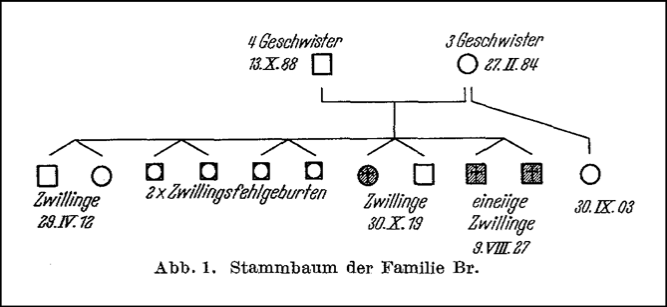

Neben den wenigen, evidenten Beschreibungen von familiären Arrhythmiesyndromen gab es natürlich auch Einzelfallbeschreibungen, insbesondere von unklaren, vorzeitigen Todesfällen, deren Ätiologie nicht bekannt bzw. eingeschätzt werden konnte und Familieninformationen nicht erhoben wurden, wo jedoch rückblickend eine Ionenkanalerkrankung vorlag. In einer sorgfältigen Aufarbeitung von Taschen („Über plötzliche, familiäre Herztodesfälle im Jugendalter“) gelang über eine Obduktion die postmortale Diagnose einer linksventrikulären Hypertrophie in einer Familie mit einer auffälligen Rate an Zwillingsgeburten und vorzeitigen Todesfällen ([75]; Abb. 3).

Der Berliner Pathologe Rössle hat sich dem Thema Zwillingsforschung und pathologisch-anatomische Auffälligkeiten durch Obduktionsstudien an Blutsverwandten in Deutschland gewidmet und damit vom Makroskopischen ausgehend einen bedeutenden Zweig der Erbforschung gelegt, wie z. B. in seinem Buch „Die pathologische Anatomie der Familie“ nachzulesen ist [61], und dabei die besondere Bedeutung der Zwillingsforschung in der Erbpathologie hervorgehoben [62], z. B. durch die Konkordanz seltener Leiden bei eineiigen Zwillingen, aber auch in der Kardioanatomie (Größe, Form, Lage, Gewicht und innerer Bau des Herzens) wie auch bei anatomischen Anomalien und Missbildungen. Es wurde u. a. postuliert, dass die pathologische Anatomie eine notwendige Ergänzung der klinischen Pathologie in der menschlichen Erbforschung ist.

Umso wichtiger ist die konsequente Aufarbeitung unklarer, plötzlicher und insbesondere frühzeitiger Todesfälle, da die nationalen Obduktions- und Sektionsraten hier im internationalen Vergleich niedrig sind [39]. In der Fortführung des initialen Gedankens von Robert Rössle wurde ein aktuelles Konsensuspapier von Vertretern aus fünf Fachgesellschatten erarbeitet, das bei solchen Fällen eine systematische Aufklärung im Stufenschema empfiehlt [65]; neben kardiopathologischen Untersuchungen entsprechend der postmortalen Stufendiagnostik sollte bei Hinweisen auf eine genetisch bedingte Herzerkrankung oder bei letztendlich unklarer Todesgenese im Einzelfall eine postmortale molekulargenetische Untersuchung („molecular autopsy“) durchgeführt werden.

## *Blick*: Gene für familiäre, arrhythmogene Erkrankungen – von der Pathophysiologie zum ersten Schritt für eine weitere, individualisierte Medizin

Im Folgenden werden exemplarisch seltene, arrhythmogene Erkrankungen aufgeführt, in denen deutsche Kardiologen und kardiogenetische Forscher einen wesentlichen Beitrag in der Aufklärung, Entschlüsselung und Genidentifizierung und pathomechanistischen Aufklärung beigetragen haben.

### Beispiel: *Jervell und Lange-Nielsen-Syndrom (JLNS) – angeborenes Long-QT-Syndrom mit angeborener Innenohrschwerhörigkeit*

Das JLNS ist extrem selten (Häufigkeit ca. 1:2000.000) und wurde in der Vergangenheit auch als kardioauditorisches Syndrom („surdo-cardiac syndrome“) bezeichnet; eine Reihe von Fällen und Familien wurden in Deutschland, Frankreich und Spanien beschrieben [1, 5, 76], nachdem die Erstbeschreibung und Namensgebung im Jahr 1957 erfolgte [36]. Das JLNS ist gekennzeichnet durch eine angeborene, hochgradige beidseitige Innenohrschwerhörigkeit und ein verlängertes QTc-Intervall, oft > 500 ms und, in Analogie zum angeborenen LQTS mit polymorphen VT (Torsade-de-Pointes) und Kammerflimmern, die sich klinisch als Synkopen, Herzstillstand oder plötzlicher Herztod äußern. Oft sind gehörlose Kinder oder Jugendliche betroffen, die unter Stress, Bewegung oder Angst synkopale Episoden haben; unbehandelt sterben viele JLNS vor dem 15. Lebensjahr. Aufgrund der schweren klinischen Ausprägung, aber auch oft unauffälligen Eltern (Hörfunktion, QTc-Dauer), wurde eine autosomal-rezessive Vererbung vermutet.

Das Münsteraner Team im Institut für Genetik von Herzerkrankungen und der damaligen Medizinischen Klinik C (Kardiologie) war bei den initialen Beschreibungen der Gene unmittelbar beteiligt: 1999 mit der Publikation, dass homozygote *KCNQ1*-Genmutationen beim JLNS (Untertyp JLN1) nachweisbar sind [16], und zuvor in der Erstbeschreibung, dass die Beta-Untereinheit desselben I_Ks_-Kanals (minK, *KCNE1*-Gen) ebenfalls ein JLNS (Untertyp JLN2) verursachen kann [70]. Heterozygote Genmutation in *KCNE1*, als Ursache des LQTS (Untertyp LQT5), sind jedoch mit einem milderen Phänotyp assoziiert, wie in einer internationalen Studie unter Einschluss der deutschen LQT5-Fälle gezeigt werden konnte: Bei LQT5-Patienten war das QTc-Intervall bei Probanden signifikant länger (476,9 ± 38,6 ms; normal: < 460 ms) als bei Genotyp-positiven Familienmitgliedern (441,8 ± 30,9 ms), resultierend jedoch in einer niedrigen EKG-Penetranz (ca. 20 %) und insgesamt niedrigen Ereignisrate für Arrhythmien (16,9 % der Probanden, nur 1,4 % der Familienmitglieder). Der LQT5-Phäntyp wird daher aktuell als mild eingestuft [60]. Die identifizierten JLNS-Genvarianten wurden in anschließenden Stammzellexperimenten (hiPSC-CM) weiter untersucht und berichtet [85].

Die Tatsache, dass es trotz klarer, im EKG identifizierbarer Risikoparameter (z. B. Länge des QTc-Intervalls) für eine Antiarrhythmika-Gabe (z. B. Klasse IC) gibt und dennoch manche Patienten ohne offensichtliche QTc-Verlängerung dennoch Torsade-de-Pointes-Tachykardien entwickeln (z. B. Chinidin-Therapie bei Vorhofflimmern > TdP-Entwicklung), ärgerte so manchen Rhythmologen, z. B. einen gewissen Professor Günter Breithardt. Er vermutete, dass hier etwas Angeborenes, ein verstecktes Long-QT-Syndrom (LQTS), dahinterstecken müsse, was nur das genetische Auge, nicht aber das kardiologische sehen könne. Also musste in Münster eine kardiogenetische Arbeitsgruppe im Jahr 1994 gegründet werden. Damals waren nur ca. 10 Patienten mit angeborenem oder erworbenem LQTS bei uns bekannt (aktuell: ca. 2400 nicht-verwandte Familien). Sein kardiologisches Auge hatte recht: In ca. 10–15 % der erworbenen LQTS-Fälle sind tatsächlich LQTS-Genmutationen *versteckt*, wie wir in einem kurzen Bericht 1997 veröffentlichten [66]. Die Kardiogenetik in Münster nahm nun seinen Lauf.

### Beispiel: *Brugada-Syndrom – ein fluenter, variabler EKG-Phänotyp*

Die genetische Basis des Brugada-Syndroms nach den Erstbeschreibungen vor über 30 Jahren [11, 47] wurde 1998 in einem internationalen Konsortium mit wesentlicher Beteiligung der Münsteraner Gruppe aufgrund einer schon damals bekannten, hohen Patientenzahl veröffentlicht: Natriumkanal-Genmutationen (*SCN5A*-Gen) als Hauptursache der Erkrankung, charakterisiert durch einen Funktionsverlust und Reduzierung der Natriumkanaldichte und/oder des Natriumeinwärtsstroms [15]. Bis heute sind dabei *SCN5A*-Genmutationen die Hauptursache der Erkrankung, in 20–25 % der Fälle; obgleich vielen andere Gene ebenfalls als ursächlich vorgeschlagen bzw. publiziert wurden, ist deren Krankheitsevidenz derzeit noch meist „limited“ oder „disputed“ (ClinGen). Die Genmutationen sind dabei innerhalb des kodierten Proteins über alle Funktionsbereiche gestreut, ohne Anreicherung für spezifische Genmutationen [38].

Die zunehmende Wahrnehmung des Brugada-Syndroms im klinischen Alltag und die Notwendigkeit zur Standardisierung (Diagnostik, Prognostik und Therapie) führte auch zur Beteiligung von Münsteraner Kardiologen an Konsensus-Konferenzen [2, 81], aber auch zu Metaanalysen zur möglichen Gewichtigkeit einer Risikoabschätzung mittels programmierter, ventrikulärer Stimulation [54] und zur Beteiligung bei der spezifischen Identifizierung von Serum-Biomarkern (*hier:* Autoantikörper gegen α‑kardiales Aktin, Keratin und Connexin-43), die zusammen mit der *SCN5A*-kodierten Natriumkanal-Untereinheit kolokalisiert sind und eine myozelluläre Schädigung zeigen [14].

### Beispiel: *Idiopathisches Sinusknotensyndrom und Erregungsleitungsstörungen – zwischen Habilitierung und erster Genbeschreibung*

Im Jahr 1978 habilitierte sich Günter Breithardt (Uniklinik Düsseldorf, später Universitätsklinikum Münster) über die „Klinisch-elektrophysiologische Untersuchungen der Sinusknotenfunktion“; er wusste damals jedoch noch nicht, dass ihn dieses Interesse später noch einmal einholen sollte, da natürlich im klinischen Alltag die ventrikulären Arrhythmien „schneller und bedeutender“ sind. Im Jahr 2003, also ein Vierteljahrhundert später, erfolgte dann erstmals aus Münster ein Fallbeschreibung, in der eine Schrittmacherpatientin mit chronotroper Inkompetenz in Ruhe genetisch untersucht wurde [69]. In einem Kandidatengen-Ansatz wurde dabei eine Hauptuntereinheit des kardialen „*pacemaker channels*“ (sog. I_f_-Kanal), das *HCN4*-Gen, analysiert und überraschenderweise eine Abbruchmutation identifiziert. Der Herzschrittmacherstrom I_f_ ist eine wichtige Determinante der diastolischen Depolarisation in Sinusknotenzellen; die Mutation führte in durch Verlust der cAMP-Bindungsdomäne in COS-7-Zellen zu einem dominant-negativen Effekt der mutanten Untereinheiten auf Wildtyp-Untereinheiten und eine reduzierte diastolische Depolarisationsfähigkeit. Die Untersuchungen wurden durch weitere Veröffentlichungen [50, 55] und auch durch andere Arbeitsgruppen international bestätigt; auch wurde eine umgekehrte Rolle des Herzschrittmacherstroms I_f_ bei Sinustachykardien postuliert.

Nach diesem ersten Finding, dass auch anderweitig nicht erklärte Sinusbradykardien (SND) und auch Erregungsleitungsstörungen genetisch bedingt sein können, wurde gezielt nach weiteren Familien mit der Erkrankung gesucht, um hier pathophysiologisch und ätiologisch weiterzukommen. Wie erwartet, war der Anteil an *HCN4*-positiven Indexpatienten gering, auch war die Analyse der verwandten Gene *HCN2* und *HCN1* nicht wegweisend.

So gelang es dem Münsteraner Team im Jahr 2019 erneut, nach Durchführung einer genomweiten Kopplungsanalyse in einer großen Familie mit SND und AV-Block (*n* = 25 Familienmitglieder) einen gekoppelten Genlocus auf dem Chromosom 7q21.1-q31.1 zu identifizieren, wo nach Kandidatengen-Analyse im *GNB2*-Gen, welches die β2-Untereinheit (Gβ2) des heterotrimeren G‑Protein-Komplexes kodiert und der bei vagaler Stimulation von G‑Protein-gekoppelten Rezeptoren, eine ursächliche Genmutation gefunden wurde. In verschiedenen, heterologen Expressionssystemen (HEK-293T-Zellen und *Xenopus-laevis-Oozyten*) war durch die Mutation der G‑Protein-aktivierte K+-Kanal (GIRK; Kir3.1/Kir3.4) über- und konstitutionell aktiv und hat wahrscheinlich somit das myozelluläre Membranpotenzial hyperpolarisiert. Die Ergebnisse beschreiben zum ersten Mal eine Rolle eines mutierten G‑Proteins in der nichtsyndromalen Schrittmachererkrankung aufgrund der Aktivierung des GIRK-Kanals [74] und betonen damit die mögliche Rolle der GIRK-Kanäle bei der Impulsgenerierung im menschlichen Sinusknoten.

Insofern war es konsequent, ebenfalls in weiteren Patienten mit familiärer oder idiopathischer Sinusknotendysfunktion (SND) in Genen für kardiale GIRK-Kanäle (Kir3.1 und Kir3.4; *KCNJ3* und *KCNJ5*) entweder in Kandidatengen-Sequenzierung oder Gesamt-Exom-Sequenzierung nach krankheitsassoziierten Genvariante zu forschen. In einer 3‑Generationen-Familie mit SND wurde dabei 2019 erstmals eine nichtsynonyme *KCNJ5*-Genmutation identifiziert, die mit dem Phänotyp kosegregierte und in heterologen Zellexpressionssystemen ebenfalls zu erhöhten GIRK-Strömen führte [43]. Die Genmutation wurde darüber hinaus jüngst von dem Team in hiPSC-CM charakterisiert [40, 41].

Bei einer Familie mit einer syndromalen Form der SND, hier in Kombination mit fokaler idiopathischer Epilepsie, konnte in einem ähnlichen Ansatz (Gesamt-Exom-Sequenzierung) eine sog. *Loss-of-function*-Genmutation in *CACNA1D *(für Cav1.3-spannungsabhängige L‑Typ-Kalziumkanäle [LTCCs]) identifiziert werden, die möglicherweise unterschiedliche, d. h. Isoform- bzw. gewebsabhängige Effekte (kardial vs. neuronal) auf das Gatingverhalten des Kanals hat, was als solches noch nicht bekannt war [59].

**Figure 4:**
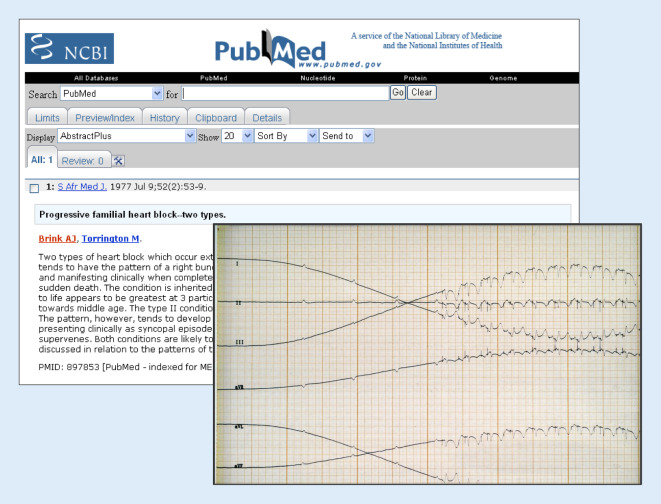

In einer großen, südafrikanischen Familie mit progressiver, autosomal-dominanter Erregungsleitungsstörung (PFHB1), eingewandert im 17. Jahrhundert auf dem Seeweg aus Portugal, war schon seit Langem bekannt, dass der ursächliche Genlocus auf dem Chromosom 19 lag [10]. In einer Kollaboration der südafrikanischen Arbeitsgruppe und dem Münsteraner Team gelang es, eine familiäre Mutation in einem transienten Rezeptorpotenzial-Kationenkanal (*TRPM4*) im chromosomalen Locus 19q13.3 zu identifizieren, die zu einem Funktionsgewinn mit erhöhter Kanaldichte durch konstitutive SUMOylierung des mutanten TRPM4-Kanals führt – eine erneute Erstbeschreibung eines Gens für diese familiäre Erkrankung, den familiären Herzblock (Abb. 4).

Die Rolle das kardialen Natriumkanal-Gens *SCN5A* für AV-Blockierungen und Brugada-Syndrom, war zuvor schon beschrieben worden; als einer der ersten familiären Fallbeschreibungen wurde 2010 vom Münsteraner Team eine homozygote Genmutation identifiziert, die im heterozygoten Fall zu keiner Phänotyp-Manifestation, im homozygoten Fall jedoch zu einer schwerwiegenden, in Jugend auftretenden kardialen Erregungsleitungsstörung führt, also einem autosomal-rezessiven Vererbungsmuster folgt [51]. Es handelte sich um eine konsanguine Familie (weißrussische Enklave); die nichtsynonyme *SCN5A*-Genmutation zeigte eine reduzierte Natriumkanalaktivierung als auch veränderte Inaktivierung, passend zum klinischen Erscheinungsbild. In humanen, induzierten pluripotenten Stammzellen aus Kardiomyozyten zeigte sich zudem, dass möglicherweise ein Switch von der adulten zur *fetalen*, weniger aktiven *SCN5A*-Isoform zugrunde liegt [78].

### Beispiel: *Short-Syndrom (SQTS) – der seltene, mitunter übersehende Counterpart zum LQTS*

Die Mannheimer und später die Münsteraner Kardiologen haben sich seit Erstbeschreibung der Erkrankung im Jahr 2000 [34] intensiv mit den klinischen Charakteristika, den Grundlagen der Erkrankung und der genetischen Basis dieser sehr seltenen Entität wissenschaftlich wie translational auseinandergesetzt.

Nach den Erstbeschreibungen im Jahr 2004, dass nichtsynonyme Mutationen im *KCNH2*-Gen [12] und im *KCNQ1*-Gen (Untertyp SQT2; [7]) mit einem sog. „gain of function“ ein SQTS verursachen können, wurde die Erkrankung weiter klinisch charakterisiert, u. a. durch elektrokardiographische wie echokardiographische Merkmale (z. B. elektromechanische Kopplung und mechanoelektrische Hypothese der U‑Welle mit Nachweis einer signifikanten Dissoziation des Endes der ventrikulären Repolarisation und der mechanischen Systole [63]) oder durch Fallbeschreibungen, dass auch ein Verlust des Cav1.2-Kanals durch eine *CACNA1C*-Genmutation ein syndromales SQTS mit extrakardialem Phänotyp (*hier:* hochfunktionaler Autismus, affektive Störung, schweren Zahnschmelzdefekten) verursachen kann [25].

Weiterhin wegweisend waren Experimente mit von menschlichen, pluripotenten Stammzellen abgeleiteten Kardiomyozyten (hiPSC-CMs) von SQT1-Patienten, bei denen der erhöhte I_Kr_-Kaliumstrom und das verkürzte Aktionspotenzial der mutanten Zellen nachgewiesen werden konnte und darüber hinaus pharmakologisch wurde [23, 86]. In transgenen Kaninchen nach Oozyten-Mikroinjektion der SQT1-Genmutation gelang es zudem, den Phänotyp der menschlichen Erkrankung bzw. der Patienten-Stammzell-Modelle mit verkürzter QT/APD und erhöhter VT/VF-Induzierbarkeit nachzubilden und den antiarrhythmischen Effekt von Chinidin durch APD-Normalisierung nachzuweisen [53]. In einer weiteren, aktuellen Arbeit wurde der Einfluss einer Mikro-RNA (miR-365) zur Regulierung repolarisierender Ionenkanäle identifiziert und eine Verlängerung des Aktionspotenzials in hiPSC-CMs beim SQT1 nachgewiesen, wohingegen spezifische Hemmung von miR-365 umgekehrt das pathologisch verlängerte Aktionspotenzial in LQT2-hiPSC-CMs durch jeweilige Regulation des repolarisierenden I_Ks_-Ionenstroms normalisiert [26].

Die klassischen Ionenkanalgene des LQTS verursachen jedoch nur in einem kleinen Anteil ein SQTS; für viele Patienten ist die Pathogenese des SQTS bzw. des assoziierten Kammerflimmerns und evtl. auch Vorhofflimmerns noch nicht aufgeklärt. Nach Identifizierung einer Genmutation in einer großen Familie mit SQTS (Untertyp SQT4) im *SCL4A3*-Gen, eines Anionaustauscher-Kanals für Chlorid und Bikarbonat [77], wurde in Kollaboration des Münsteraner Genetik-Teams mit den Kopenhagener Kollegen klar, dass dieses Gen derzeit das Hauptgen für SQTS ist mit einem relativen Anteil von 20–25 % und somit einen wichtigen Baustein in der leicht übersehbaren Arrhythmieform („idiopathisches Kammerflimmern“) darstellt [18].

## *Seitenblick*: Gene für familiäre Kardiomyopathien

Nicht nur im Bereich der hereditären Arrhythmien, sondern auch bei den Kardiomyopathien erfolgten wichtige Publikationen aus Deutschland, die durch die Identifizierung von Hauptgenen einen Meilenstein im Verständnis und bei der Diagnose der Erkrankungen darstellen und deswegen im Folgenden ebenfalls kurz erwähnt werden. Maßgeblich war die Arbeitsgruppe von Ludwig Thierfelder (damals Berlin) beteiligt.

Die *arrhythmogene rechtsventrikuläre Kardiomyopathie* (ARVC; früher auch ARVD: „Dysplasie“) ist charakterisiert durch einen zunehmenden Verlust von Kardiomyozyten und den Ersatz durch Fett- und Bindegewebszellen, insbesondere im rechten Ventrikel, aber auch biventrikulär oder linksseitig dominant (ALVC), weswegen auch der Begriff „arrhythmogene Kardiomyopathie“ (ACM) eingeführt wurde [20]. Die klinische Manifestation sind typische EKG-Veränderungen (z. B. Epsilon-Potenzial, T‑Wellen-Negativierungen, VES), ventrikuläre Tachykardien, Herzinsuffizienz und plötzlicher Herztod; erste Fall- und Serienbeschreibungen erfolgten insbesondere in Frankreich und Italien [28, 46]. Dass es sich bei der ARVC überwiegend um eine Desmosomen-Erkrankung, d. h. genetische bedingte Defekte in Adhäsionsproteinen der Zellmembran zur myozytären Verankerung der Zellen (Zell-Zell-Kontakt) und mechanischen Stabilisierung mit Intermediärfilamenten (Zytoskelett) handelt und dass heterozygote Mutationen im desmosomalen Protein Plakophilin‑2 (Genname: *PKP2*) eine wesentliche Ursache der autosomal-dominant vererbten Form der ARVC sind, wurde erstmals 2004 publiziert [31]; das Gen *PKP2* ist auch heute noch Hauptursache der ARVC (ca. 30–40 %), und der Nachweis einer pathogenen Genmutation ist mittlerweile in den TaskForce-Kriterien ein Majorkriterium bei der Diagnosestellung [20]. Zuvor war lediglich bekannt, dass sehr seltene, rezessive und syndromale Formen der ARVC auf biallelische Genmutationen (Plakoglobin-Gen *JUP*: Naxos-Syndrom, ARVC + Keratome + wolliges Haar [48, 83]; *DSP*-Gen: Carvajal-Syndrom, DCM + Keratome + wolliges Haar [52]), und damit Komponenten des Desmosoms mechanistisch bei der ARVC eine Rolle spielen können.

Auch bei der *dilatativen Kardiomyopathie (DCM)* wurden bereits 1949 familiäre Formen beschrieben, die zunächst als *idiopathisch* klassifiziert wurden [27]. Weitere, familiäre Beschreibungen von Kardiomyopathien folgten [32, 80] und unterstützten dabei die Entwicklung einer Klassifikation von Kardiomyopathien, erstmals durch die International Society and Federation of Cardiology (ISFC; [5]) und aktuell durch die ESC [84], wobei es zunehmend nicht nur um die Differenzierung der Polyätiologie einzelner Kardiomyopathien geht, sondern auch um eine effiziente Diagnostik, Therapie und Risikostratifizierung, unter Einschluss kardialer Bildgebungsparameter und ursächlicher, genetischer Faktoren, wie z. B. in den „Risiko-DCM-Genen“ *LMNA, PLN, FLNC* und *RMB20* [42]. Die ursprünglichen Kardiomyopathien haben somit ihre *idiopathische Genese* [21, 64] weitgehend verlassen, obgleich die Mutationsdetektionsrate bei den einzelnen Kardiomyopathie-Formen lediglich zwischen 20 und 60 % liegt. Im Hinblick auf die DCM war ein wesentlicher, wissenschaftlicher Erfolg diesbezüglich die Erstbeschreibung und Identifizierung von Loss-of-function-Genmutationen im Titin-Gen auf dem Chromosom 2q31 durch die deutsche Arbeitsgruppe [30]; das Gen *TTN* kodiert das größte, humane Protein (auch „Connectin“; ca. 3,6 MD, > 34.000 Aminosäuren mit 320 Proteindomänen) und ist ein Intermediärfilament-Protein der quergestreiften Muskulatur, welches Myosinköpfe zwischen den Aktinfilamenten während der Myokardkontraktion stabilisiert und zentriert. Der Name Titin ist dabei eine Abkürzung der chemischen Verbindung „*Methionylthreonylthreonylglutaminylalanyl … isoleucin*“, die zudem das längste Wort der Welt mit 189.819 Buchstaben ist. Die Erstautoren fanden dabei heraus, das *TTN*-Genmutationen eine autosomal-dominante DCM verursachen und in ca. 25 % bei anderweitig *idiopathischer DCM* nachweisbar sind. Mittlerweile wurden auch TTN-Genmutationen in Frühformen des idiopathischen Vorhofflimmerns („early onset atrial fibrillation“) identifiziert [17, 35], am ehesten als Ausdruck einer atrialen Kardiomyopathie.

In einer weiteren Studie wurden Patienten mit dem sog. Chromosom 1p36-Deletionssyndrom (d. h. einer partiellen Monosomie mit u. a. fazialen Dysmorphien, Muskelhypotonie, Entwicklungsverzögerung, reduzierter Intelligenz, Krampfanfällen, Herzfehlern, Schwerhörigkeit und pränatalem Kleinwuchs) klinisch und genetisch untersucht, wenn zusätzlich eine DCM bzw. eine linksventrikuläre Non-Compaction als Phänotyp auftrat [4]; das Gen *PRDM16*, ein Zink-Finger-Transkriptions-Koregulator, welches im relevanten Chr. 1p36-Bereich liegt, wurde bei 18 Patienten mit einem zusätzlichen, kardialen Phänotyp als betroffen identifiziert; in einer unabhängigen Kohorte von 75 Patienten ohne Chr. 1p36-Deletion zeigte eine Sequenzanalyse, dass überwiegend Loss-of-function-Genvarianten ca. 3–5 % von Patienten mit DCM/LVNC nachweisbar sind, die in Kontrollen sehr selten sind [4]. PRDM16 wurde zuvor nicht mit Herzerkrankungen, sondern primär mit hämatoonkologischen Erkrankungen, in Verbindung gebracht; eine induzierte *PRDM16*-Haploinsuffizienz im Zebrafisch führte zu einer kontraktilen Dysfunktion, partiellen Entkopplung von Kardiomyozyten und reduzierten Kardiomyozyten-Proliferationskapazität [4], im Mausmodell (Knock-out) zu einer altersabhängigen Herzhypertrophie, kardialem Remodelling, mitochondrialen Dysfunktion und Herzinsuffizienz [19], was eine physiologisch relevante Rolle von *PRDM16 *unterstreicht.

## *Rundblick*: Genetische Kovarianz – ein weiterer Schlüssel und mögliches Add-on für phänotypische (interindividuelle) Variabilität

Schon seit den ersten Familienbeschreibungen, aber auch in der klinischen Queranalyse von Patienten mit derselben Grunderkrankung (z. B. durch verschiedene, aber funktionell ähnliche Mutationen im selben Krankheitsgen) oder und auch mit demselben Genotyp (z. B. Familienmitglied) war für Arrhythmien, aber auch für Kardiomyopathien evident, dass eine erheblich unterschiedliche, inter- wie auch intrafamiliäre Krankheitsmanifestation und Krankheitsausprägung bestehen, d. h. eine inkomplette, zum Teil altersabhängige Erkrankungspenetranz und phänotypische Variabilität. Neben der kausalen, monogenen Ursache und damit Krankheitsprädisposition, z. B. für eine spezifische Arrhythmie- oder Kardiomyopathie-Form, gibt es zusätzliche, individuelle Krankheitsmodifikatoren, die einerseits exogen (z. B. Lifestyle, körperliche Aktivität …), andererseits endogen (Geschlecht, Hormonstatus, Familienanamnese, zusätzliche genetische Kovarianz …) sind. In Bezug auf genetische Kovarianz gibt es zusätzlich zu den pathogenen Genvarianten (Mutationen, selten, oft < 0,05 % in Populationsdatenbanken [z. B. gnomAD]; meist familienspezifisch) und ihrer Anzahl polymorphe Genvarianten mit einem intermediären Effekt (Allelfrequenz in Kontrollen < 1 %), die jedoch in bestimmten Erkrankungsgruppen (z. B. HCM-Patienten) häufiger sind als in der generellen Bevölkerung, und häufigere, polymorphe Genvarianten (> 1 % in Kontrollen), die sich über additive Aspekte (z. B. polygener Risiko-Score) und in multipler Kombination klinisch auswirken können.

Verschiedene, kardiogenetische Arbeitsgruppen in Deutschland haben sich daher dem Thema „Genomweite Assoziationsstudien“ im Bereich der Arrhythmien, Kardiomyopathien und natürlich der Lipidstoffwechselstörungen gewidmet. Im Folgenden werden exemplarisch einige federführende Beispiele genannt; zusätzlich gibt es viele, hochrangige Publikationen auf der Basis internationaler Kollaborationen mit Beteiligung der deutschen, kardiogenetischen Arbeitsgruppen.

Genomweite Assoziationsstudien (GWAS) zielen darauf ab, solche häufigen, genetische Varianten zu identifizieren, die mit Merkmalen und Krankheiten durch den Vergleich großer Patienten- und Kontrollgruppen assoziiert sind. Seit 2005 wurden mehr als 5000 GWAS für fast ebenso viele Merkmale veröffentlicht. Diese Studien haben Einblicke in die chromosomale Loci und ihre Gene gegeben, die phänotypischen Merkmalen zugrunde liegen, haben genetische Korrelationen zwischen Merkmalen und Krankheiten hervorgehoben und beginnen, den klinischen Nutzen zu demonstrieren, indem sie Personen mit erhöhtem Risiko für häufige Krankheiten identifizieren [6, 8, 44, 79]. GWAS werden häufig bei Herz-Kreislauf-Erkrankungen und damit verbundenen phänotypischen Merkmalen eingesetzt, wobei die Erkenntnisse durch multizentrische Registerstudien und große Biobank-Datensätze erleichtert werden. Ziel ist es, genregulatorische Netzwerke mit möglicher Phänotypmodulation zu identifizieren, z. B. „expression quantitative trait loci“ (eQTL) oder „splicing quantitative trait loci“ (sQTL) oder durch RNA-Sequenzierungstechnologien eine Quantifizierung der allelischen Expression (Mutant vs. Wildtyp).

Eine erste, wegweisende Publikation erfolgte 2009 mit dem Ziel, anhand von GWAS-Daten aus fünf großen, europäischen Populationskohorten (ARIC, KORA, SardiNIA, GenNOVA und HNR) neben dem bekannten NOS1AP-Genlocus weitere Loci zu identifizieren, die statistisch signifikant das QT-Intervall modulieren. Es konnten hierbei 4 Genloci im Bereich bekannter LQTS-Gene statistisch signifikant identifiziert und erneut bestätigt werden [56], ferner noch fünf weitere Loci, wo im Weiteren bislang keine monogenen Ursachen für ein LQTS oder SQTS berichtet wurden [57]. In zwei analogen Folgearbeiten [58, 72] der Münchner Arbeitsgruppe wurden einerseits Genloci identifiziert, die das PR-Intervall modulieren (insgesamt 9 chromosomale Loci mit einem *p*-Wert < 5 × 10^−8^, u. a. an den Genloci für die spannungsabhängigen Natriumkanalgene *SCN10A *und *SCN5A*, ferner für kardiale Entwicklungsgene wie *NKX2‑5, SOX5, WNT11* und *TBX5-TBX3*), die zum Teil pathophysiologisch analog ebenfalls mit Vorhofflimmern assoziiert sind (z. B. *SCN5A, SCN10A, NKX2‑5, SOX5*) und andererseits das QRS-Intervall (z. B. *SCN10A*-Locus).

Ein weiterer Schwerpunkt mit beachteten Publikationen lag neben der Identifizierung von Genloci für bestimmte EKG-Parameter in der Etablierung von Loci für Vorhofflimmern; so wurden vor über 20 Jahren erste Genloci beschrieben, die mit Vorhofflimmern assoziiert sind [24] (Intronvariante im *KCNN3*-Gen, an der atrialen Repolarisation beteiligt; ferner auch [71]), aber auch auf einem chromosomalen Bereich des Chr. 4q25 [37] und die damit vorherige Ergebnisse von deCODE genetics bestätigten [33].

In Bezug auf Kardiomyopathien wurde in einer weiteren GWAS ein Locus auf dem Chr. 6p21 identifiziert [49], der durch den Vergleich von 4100 DCM-Fällen und 7600 gematchten Kontrollen möglicherweise ein sog. eQTL darstellt und wiederum in einer unabhängigen Kohorte repliziert werden konnte. Unter Ausnutzung von expressionsquantitativen Merkmalsloci (eQTL) als molekulare Phänotypen identifizierten wir rs9262636 als eQTL für mehrere nahe beieinanderliegende Gene, die Klasse 1- und Klasse 2 2-MHC-Gene kodieren.

Zum jetzigen Zeitpunkt sind polymorphe Genvarianten mit einem intermediären Effekt (Allelfrequenz in Kontrollen < 1 %) oder häufigere, polymorphe Genvarianten (> 1 % in Kontrollen) noch nicht im klinischen Alltag zur Risikobeurteilung eines Erkrankungsstatus implementiert, weil sie im Vergleich zur herkömmlichen und leicht erhebbaren Parametern im EKG oder TTE in ihrer Gewichtigkeit unterlegen sind.

## *Ausblick*: Etablierung und Implementierung von kardiogenetischen Leitlinien – Begleiter für Ärzte und Patienten im medizinischen Alltag

Die Erkenntnis und Kenntnis, dass eine Vielzahl meist seltener kardiovaskulärer Krankheiten eine genetische Ursache hat und in Abhängigkeit der genetischen Ursache nicht nur die Diagnose als solche, sondern auch die Prognose der dahinterliegenden Erkrankungen beeinflusst wird, hat zur Formulierung von klinischen Empfehlungen und Leitlinien auch im deutschen, kardiologischen Fachgebiet geführt. So wurde *2015 das erste nationale Positionspapier zur Genetik kardiovaskulärer Erkrankungen* verfasst, welches eine Zusammenarbeit der Deutschen Gesellschaft für Kardiologie (DGK) und der Deutschen Gesellschaft für Pädiatrische Kardiologie (DGPK) darstellt [67]; die Themen des Papiers waren spezifische Arrhythmieformen, Kardiomyopathien und auch Lipidstoffwechselstörungen und entsprechende Empfehlungen, wann, wie eine Genotypisierung unter welchen Aspekten (diagnostisch, prognostisch) erfolgen sollte. Aufgrund der genetischen Determinierung und damit der Möglichkeit der Vererbung der Erkrankungen war klar, dass immer auch eine über den Indexpatienten hinausgehende Betrachtung erfolgen sollte, die unter Berücksichtigung des Erbmodus (von monogen: autosomal dominant bis rezessiv, bis polygen und multifaktoriell) auch immer die klinische und ggf. genetische Untersuchung von biologisch verwandten Familienmitgliedern (sog. Kaskaden-Screening) miteinschließen sollte. Häufig wird die Verpflichtung bzw. Aufgabe des Arztes, als Teil der Anamneseerhebung sich so eingehend wie nur möglich über die Familienanamnese seines Patienten zu orientieren, nur bedingt erfüllt, obgleich dieses klinisch von unschätzbarem Wert sein kann – sowohl diagnostisch-ätiologisch als auch präventiv-prognostisch gen familiären Genträgern.

Mit dem Gesetz über genetische Untersuchungen bei Menschen (Gendiagnostikgesetz – GenDG; in Kraft getreten am 1. Februar 2010) in Deutschland wurden zudem Qualifikationsinhalte für die genetische Beratung im Rahmen genetischer Untersuchungen veröffentlicht, die zuvor nur ausschließlich in den Weiterbildungsordnungen zum Facharzt für Humangenetik sowie in der Zusatz-Weiterbildung „Medizinische Genetik“ geregelt waren. Hiernach wurden im Rahmen der sog. „Qualifikation zur fachgebundenen humangenetischen Beratung“ Inhalte und Anforderungen für Ärztinnen und Ärzte, die nicht die Bezeichnung Fachärztin oder Facharzt für Humangenetik oder die Zusatzbezeichnung Medizinische Genetik führen, definiert, damit diese selbst im entsprechenden Fachgebiet eine genetische Beratung im Rahmen von diagnostischen und prädiktiven genetischen Untersuchungen durchführen können. Dieses ermöglicht somit auch (kinder)kardiologischen Kollegen, auf der Basis ihrer klinischen Kenntnisse und Erfahrung mit den seltenen Krankheitsbildern, eine zusätzliche und oft indizierte molekulargenetische Diagnostik durchzuführen. Derzeit ist jedoch noch unklar, wie viele Kardiologen und Kinderkardiologen eine solche Zusatzqualifikation erlangt haben.

Aufgrund der rapiden Entwicklung der Sequenziertechnologien (Next-Generation-Sequencing, NGS) und der zunehmenden Erkenntnis von der genetischen Pathogenitätsevidenz von Genen und ihren Genvarianten wurde eine Überarbeitung des Positionspapiers aus dem Jahr 2015 notwendig. Im Jahr 2023 erfolgte konsequenterweise in Form eines Konsensuspapiers ein Update des kardiogenetischen Fachwissens [68], an dem letztendlich drei Fachgesellschaften (DGK, DGPK und Deutsche Gesellschaft für Humangenetik [GfH]) interdisziplinär beteiligt waren. Das Ziel der Zusammenarbeit ist es, für die entsprechenden, seltenen Herzerkrankungen Diagnostik, Beratung und Therapie in der Kardiologie und Kinderkardiologie durch zunehmendes Spezialwissen und humangenetische Kenntnisse zu verbessern, um eine integrierte, interdisziplinäre Versorgung von Familien mit erhöhtem, plötzlichen Herztodrisiko zu ermöglichen.

Das Positionspapier aus 2015 wie auch das aktuelle Konsensuspapier und damit die Bedeutung genetischer Untersuchungen im Hinblick auf plötzliche Herztodesfälle wurde im Weiteren durch die Veröffentlichung des *Konsensuspapiers „Postmortale Molekulargenetische Untersuchungen (Molekulare Autopsie) bei kardiovaskulären und bei ungeklärten Todesfällen“* unterstrichen, welches ebenfalls ein Novum ist und erstmalig 2021 in Deutschland formuliert wurde; es stellt ein Konsensuspapier der Deutschen Gesellschaft für Kardiologie (DGK), der Deutschen Gesellschaft für Pädiatrische Kardiologie (DGPK), der Deutschen Gesellschaft für Humangenetik (GfH), der Deutschen Gesellschaft für Pathologie (DGP) und der Deutschen Gesellschaft für Rechtsmedizin (DGRM) dar. In diesem Papier wird ein besonderer Fokus auf die Aufklärung möglicher Ursachen eines plötzlichen, unklaren Herztodes im Rahmen einer postmortalen Stufendiagnostik (Obduktion inkl. Toxikologie und Histopathologie > Kardiopathologie > Molekulare Autopsie bzw. postmortale molekulargenetische Untersuchungen) dargestellt und im Einzelfall empfohlen (Klasse IIB−C), um einerseits die Sensibilität für Obduktionen in Deutschland zu erhöhen und somit die Dunkelziffer unklarer oder nichtuntersuchter/aufgeklärter, frühzeitiger Todesfälle zu reduzieren. Bei etwa der Hälfte der plötzlichen oder unklaren Todesfälle bei Kindern, Jugendlichen oder Erwachsenen unter 40 Jahren – sei es zum Beispiel ein plötzlicher Kindstod, Badetod, Sportlertod oder unerwarteter nächtlicher Tod – könnte eine genetisch bedingte Erkrankung des Herzens wie eine Kardiomyopathie oder ein angeborenes Arrhythmiesyndrom vorliegen – was im Fall eines genetisch bedingten Todesfalls für verwandte Familienmitglieder ebenfalls klinisch wie präventive Konsequenzen hätte [39].

Die genannten nationalen Empfehlungen sind in weitestgehendem Einklang mit Empfehlungen und Einschätzungen übergeordneter Fachgesellschaften; zudem sind deutsche Kardiologen an aktuellen, internationalen Empfehlungen (2022: „European Heart Rhythm Association [EHRA]/Heart Rhythm Society [HRS]/Asia Pacific Heart Rhythm Society [APHRS]/Latin American Heart Rhythm Society [LAHRS]: Expert Consensus Statement on the state of genetic testing for cardiac diseases“; 2023: „ESC Guidelines for the management of cardiomyopathies“) beteiligt [3, 82].

Blickt man zurück auf die deskriptiven, historischen Beschreibungen von familiären Fällen mit Arrhythmien oder Kardiomyopathien, die für sich meist seltener sind, so hat sich durch die zunehmenden, wissenschaftlichen Erkenntnisse aus den familiären Fallbeschreibungen eine wissenschaftlich solide Basis gebildet, die durch die Anwendung von hochmoderner Sequenziertechnik zu einer ätiologisch genetischen Aufklärung geführt hat. Die Kenntnis des ursächlichen Gens und seiner pathogenen Variante, nebst Entschlüsselung des ursächlichen Mechanismus im Krankheitskontext, zeigen, wie sehr die klinischen Familienbeschreibungen wertvoll für eine noch weiter personalisierte Medizin waren und immer noch sind: Sie haben die Tür für die Identifizierung neuer Gene, ihren *Pathways* und direktiven Therapien geöffnet, die versuchen, mechanistisch-ursächlich die myozelluläre Dysfunktion aufzunehmen und zu normalisieren (z. B. Mavacamten und HOCM). Die Erkenntnisse der letzten 30 Jahre zeigen zudem, dass die klare phänotypische Erfassung und der „Blick auf das Ganze“ (Patient, Klinik und Familienanamnese) gestern, heute wie morgen aktuell sind und die weitere Medizin – mit und insbesondere gemeinsam mit dem Patienten und seiner Familie – nachhaltig prägen werden.
